# Shifting linkages: Agro-pastoralism changes in the Upper Spiti Landscape and the emerging role of free-ranging dogs

**DOI:** 10.1007/s13280-026-02390-2

**Published:** 2026-05-03

**Authors:** Chandrima Home, Siddhartha Krishnan, Yash Veer Bhatnagar, Abi Tamim Vanak

**Affiliations:** 1 Shristi Manipal Institute of Art, Design and Technology, Manipal Academy of Higher Education, Manipal, India; 2 Ashoka Trust for Research in Ecology and the Environment, Royal Enclave, Sriramapura, P.O. Jakkur, Bangalore, 560064 India; 3 Centre for Doctoral Studies, Manipal Academy of Higher Education, Manipal, India; 4 International Union for Conservation of Nature, H-4, 2nd Floor, Green Park Extension, New Delhi, 110016 India; 5 Nature Conservation Foundation, Mysore, India; 6 School of Life Sciences, University of KwaZulu-Natal, Durban, South Africa

**Keywords:** Agricultural change, High altitude desert, Human-subsidized carnivore, Livestock change, Socio-ecological systems

## Abstract

The Himalayan mountains have been going through a series of ecological and social transformations. In systems where communities depend on natural resources, externalities in government policies and development interventions can have unexpected consequences for people–environment relationships. Our study investigates agro-pastoralism changes across a decade in the Upper Spiti Landscape in Himachal Pradesh, India. It evaluates these changes in the framework of ecological and social perturbations with respect to human–dog relationships. We compared livestock population trends across a temporal scale using interviews and secondary data. Our results indicate a decline in livestock population across the years from 2003 to 2013, specifically a reduction in small-bodied livestock due to dogs. The study highlights the changing agriculture-livestock nexus with increasing demands for manure from outside. Dogs in the landscape have emerged as disrupters influencing the intricately linked production systems. The research reiterates the need for concerted efforts by multiple agencies for dog population management.

**Supplementary Information:**

The online version contains supplementary material available at https://doi.org/10.1007/s13280-026-02390-2.

## Introduction

The Himalayan mountains have been going through a series of ecological and social transformations. The basic features of the resource base and production systems are “mountain specificities,” whose key characteristics include inaccessibility, fragility, marginality, and niche, as well as human adaptation mechanisms (Jodha et al. 1992). These specificities influence the pace and nature of change in mountain areas, determining the significance and effectiveness of interventions brought about by globalization (Kreutzmann 1995; Jodha 2005). While mountain societies are generally characterized by diversification, dynamism, and flexibility in their production systems (Bishop 1998), they are susceptible to change. Currently, these mountain systems are facing livelihood challenges imposed by intrinsic and extrinsic factors (Shukla et al. 2018), resulting in compromised critical ecosystem services and increased vulnerability, which in turn affects human well-being (Klein et al. 2019). In a system where communities depend on natural resources, externalities in the form of government policies and development interventions can have unexpected consequences, thereby altering people’s interaction with their environment.

The last three decades in the Spiti region of the Trans-Himalayas have witnessed a series of socio-economic transitions (Mishra 2000; Mishra et al. 2003; Singh et al. 2015), and consequent changes in the production systems and economies of the agro-pastoralist communities. Livestock rearing is essential to their livelihoods, as the manure from these production systems is utilized in agriculture. Both livestock rearing and cultivated crops are important land-use practices with the highest economic value (Murali et al. 2017). The livestock production system in the landscape comprises large, free-ranging species, such as yaks and horses, as well as medium- to small-bodied species like yak-cow hybrids (*dzomo*, *dzo*), cattle, donkeys, sheep, and goats (Mishra et al. 2003; Suryawanshi et al. 2013).

In the mid-1980s, agro-economic changes with the introduction of green peas (*Pisum sativum*) as a cash crop, and increased tourism after 1991, helped integrate local economies with regional markets (Mishra 2000). Climatic changes in the last decade have resulted in flourishing apple cultivation in the valley’s lower reaches, opening the local economy to national and international markets (Rana et al. 2012; Basannagari and Kala 2013). In 2002, a government scheme (*Nautod*) helped provide land to the landless and marginal farmers. Between 1998 and 2008, agricultural land increased by approximately 15% through this scheme (Anonymous 2011). Development schemes under several government agencies (Employment guarantee schemes, universal education, agri-schemes from NABARD), and tourism, have also brought greater employment opportunities in the region (Anonymous 2011; Singh et al. 2015).

Over the years, while the growth in tourism infrastructure has supported livelihoods, the absence of proper waste disposal mechanisms has resulted in piles of garbage, supporting a large and stable domestic dog population (Pal 2013; Home et al. 2017). This has resulted in new forms of human-animal conflict, with an increasing trend of dog-bite cases (Home, unpublished), and dogs becoming increasingly feral and depredating livestock (Suryawanshi et al. 2013; Home et al. 2017; Home 2018). While agro-pastoralists here have always dealt with losing livestock to wild carnivores (Bagchi and Mishra 2006), the additional pressure of losses to dogs has been noteworthy. Nearly 80% of depredations by dogs comprised small-bodied livestock, and the economic losses to dogs were as high as those lost to wild carnivores (Home et al. 2017).

In this study, we aim to analyze the agro-pastoral changes and understand the interactions across a temporal scale, specifically within the context of livestock depredation by dogs. Since livestock rearing is an indispensable component of agro-pastoral livelihoods, changes in livestock population and their management over time can affect these linkages in several ways. Specifically, we intend to (1) identify and analyze the trends in livestock population size and composition for the timeline 2003–2013, and identify reasons for livestock changes, (2) understand how these changes have been affecting the agricultural inputs, and (3) discuss possible future livestock trends in the context of livestock depredation by dogs on existing agro-pastoral linkages.

## Materials and methods

### Study area

The study was conducted in the Upper Spiti Landscape (USL), an administrative unit within Spiti, a subdivision of the Lahaul and Spiti District in Himachal Pradesh. The area experiences frigid winters (~ − 40 °C in peak winter) while summer temperatures range from 4 to 30 °C, and precipitation mostly occurs as snow. The area is part of the Tibetan Plateau and has the lowest human density in the country (2 persons/sq. km)^1^. The Spitians are mostly Buddhists and follow the Tibetan and Bhoti cultures. This plateau supports transhuman-pastoralism, agro-pastoralism (Bishop 1998), and a unique assemblage of wild fauna (Mishra 1997; Anonymous 2011).

Thirty villages within the study area are distributed across eight *panchayats* (a term used to describe a village council) spread on either bank of the Spiti River. The sub-divisional headquarters, Kaza, and 22 villages are located along the National Highway (NH505) that traverses the valley bottom, providing better accessibility compared to the eight villages that are situated away from the river (as depicted in Fig. 4). While the side valleys have pastures in pockets, the plateau has relatively continuous pastures.

### Data collection and analysis

We conducted semi-structured interviews across 27 villages in eight panchayats from October 2014 to January 2015. A convenience sampling framework was adopted (Robinson 2014), and nearly 30–40% of the households were targeted in each village. The questionnaire survey was designed for two specific objectives; (a) to collect responses on explicit questions designed to capture attitudes of respondents toward dogs (Home, unpublished data; Home 2018) and (b) to engage in understanding the larger changes in livestock population and livestock depredation by dogs in the context of existing production systems and their linkages (See Supplementary Document for Questionnaire).

In each household, one adult was interviewed based on their availability. However, care was taken to ensure balanced responses across genders. Before conducting the interview, the respondent was provided with a brief background on the research being conducted by the interviewer (CH) with consent to participate. The Ashoka Trust for Research in Ecology and the Environment granted ethical approval for this study, as indicated in the approval letter number IRB/ACA/0015/CH/2009X. Each interview lasted an average of 22 min and was recorded with the respondent’s prior permission. The questionnaire was administered mainly in Hindi (CH) and/or Spitian language (by a local field assistant) when required. Detailed field notes made during informal conversations with residents and group discussions with village elders further aided the triangulation of data collected through face-to-face interviews.

Before data collection, a pilot survey was carried out (August 2013–December 2013), where 68 respondents were interviewed across 12 villages. The pilot survey played a crucial role in refining the questionnaire for subsequent data collection (October 2014–January 2015). The section designed to engage respondents to understand the production systems and the linkages allowed a mix of closed and open-ended responses for gathering information on the following specific aspects:

1. Changes in the livestock population across 2003–2013, both in terms of number, composition, and perceived reasons for change
2. Effects of livestock population change on agriculture in terms of manure inputs
3. Perception of livestock damage by dogs and preferred future livestock composition

We compared the livestock population in the landscape across three temporal zones (1987, 2003, and 2013), which spanned nearly two and a half decades. For the years 1987 and 2003, we used livestock holding data published in the management plan (Anonymous 2011). Key informant interviews with herders conducted from January to February 2014 provided data on the livestock population for the year 2013. The Upper Spiti Valley has been the focus of extensive research and conservation efforts for the last two decades (Nature Conservation Foundation in partnership with government and non-government agencies). The existing long-term relationship helped establish a record-keeping system and identify the key herders in the villages. Livestock holding across the years has been represented as sheep units (SU) based on relative forage consumption (Foose 1982).

All interviews were transcribed into English. From the questionnaire survey (See Supplementary Information), the following variables were considered deductive in nature; livestock population trends (increase/ decrease/ no change/ unsure), presence/absence of small-bodied livestock, access manure from outside (yes/no), sell manure (yes/no), whether respondent would maintain the same livestock composition in the future (yes/no), experience of livestock depredation by dogs (yes/no). The binary responses for these variables were coded as 0 or 1. In the case of open-ended questions that encouraged respondents to mention reasons for livestock decline, changes in livestock composition, reasons for future livestock preferences, and access to manure, several themes emerged. Each theme was coded with a value of 0 or 1, depending on the responses received. For example, respondents were free to mention more than one reason for livestock decline, and the themes that emerged were coded accordingly. We present the coded data as percentages. In cases where multiple reasons can be expressed, percentages did not add up to 100.

## Results

Of the 257 interviews conducted across 27 villages, we present the results for 256 semi-structured interviews (males, 56%; females, 44%; mean age, 46 years). We discarded one interview due to inadequate information.

### Changes in livestock population and perceived reasons for change

Nearly 72% of respondents reported a decline in livestock numbers between 2003 and 2013, followed by 16% who reported no change, 10% who reported an increase, and 2% who were unsure about the change in livestock numbers. Nearly 59% of the respondents did not have small-bodied livestock, and 40% of these (out of the 59%) experienced this reduction in the last 5 years (2009–2014). About a quarter of respondents had not reared small-bodied stock for over a decade, and an additional 31% stopped rearing them in the past decade (2003–2013). While a few villages along the well-connected Spiti valley (Kaza, Rangrik, Khurik, Sumling, and Hull) had stopped rearing small livestock for the last five to 10 years or even more (especially Kaza and Rangrik), post 2011, six villages in the upper reaches of the valley (Gete, Tashigang, Lidang, Pangmo, and recently Lara and Shego in 2016) decided to stop rearing small livestock. The villages that still maintained sheep and goat populations reported a 50–75% decline in the number of households owning them over the last 10 years. About 7% of the respondents kept horses primarily for cultural occasions.

**Fig. 1:**
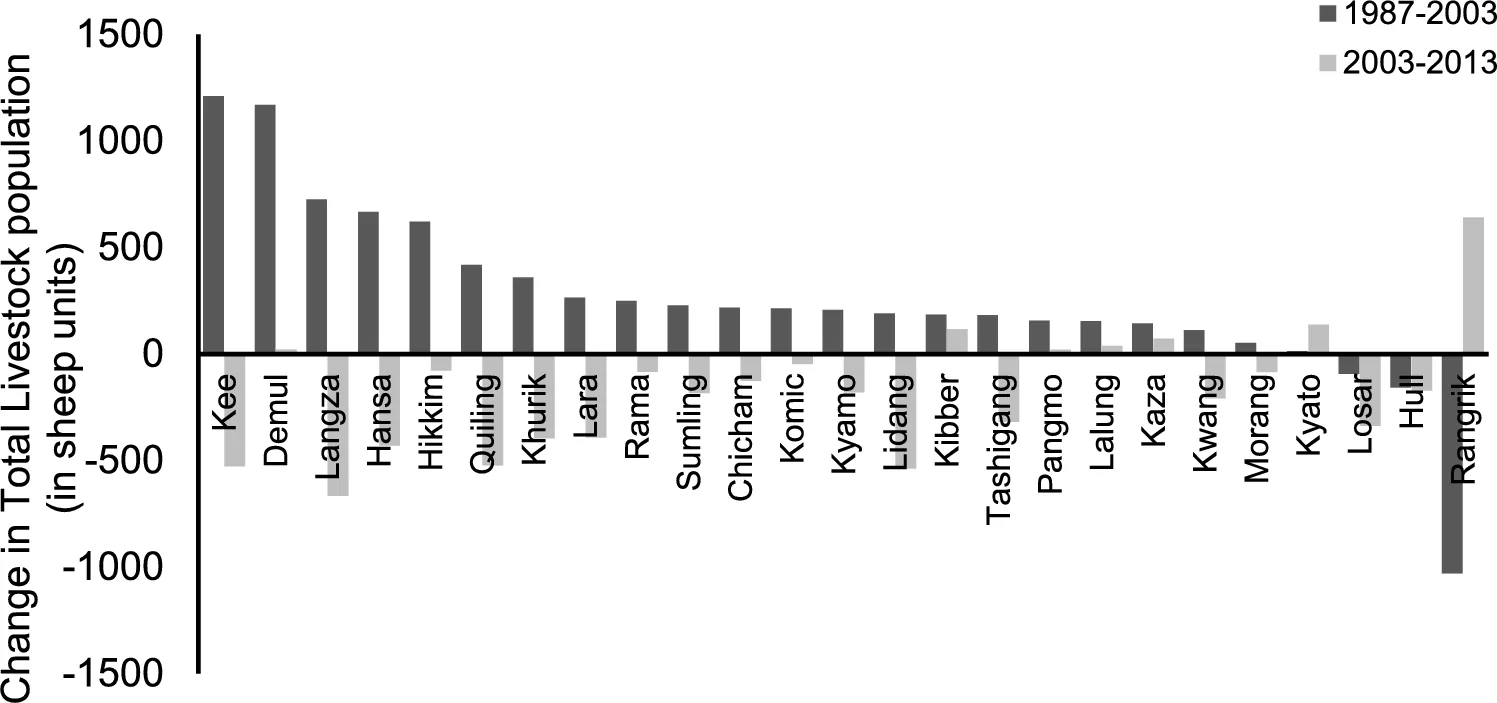
Change in total livestock population (in sheep units) from 1987 to 2003 and from 2003 to 2013 (*N* = 25 villages). *Source*: Census data for 1987 and 2003 (courtesy Nature Conservation Foundation) and information from damage census data for 2013. Based on forage consumption, livestock abundance has been depicted in Sheep Units (Foose 1982)

Livestock population trends compared across a larger temporal scale showed an increase in overall livestock population between 1987 and 2003, followed by a decline after 2003 (Fig. 1). Data comparisons revealed a 44% decline in livestock population over the last decade (*N* = 25 villages, 2003–2013). In terms of composition, post-2003, we observed a reduction in the population of small-bodied livestock (71% decline in sheep, 68% decline in goat population) and larger livestock such as horses (16% further reduction from the 1987–2003 decline observed).

**Fig. 2:**
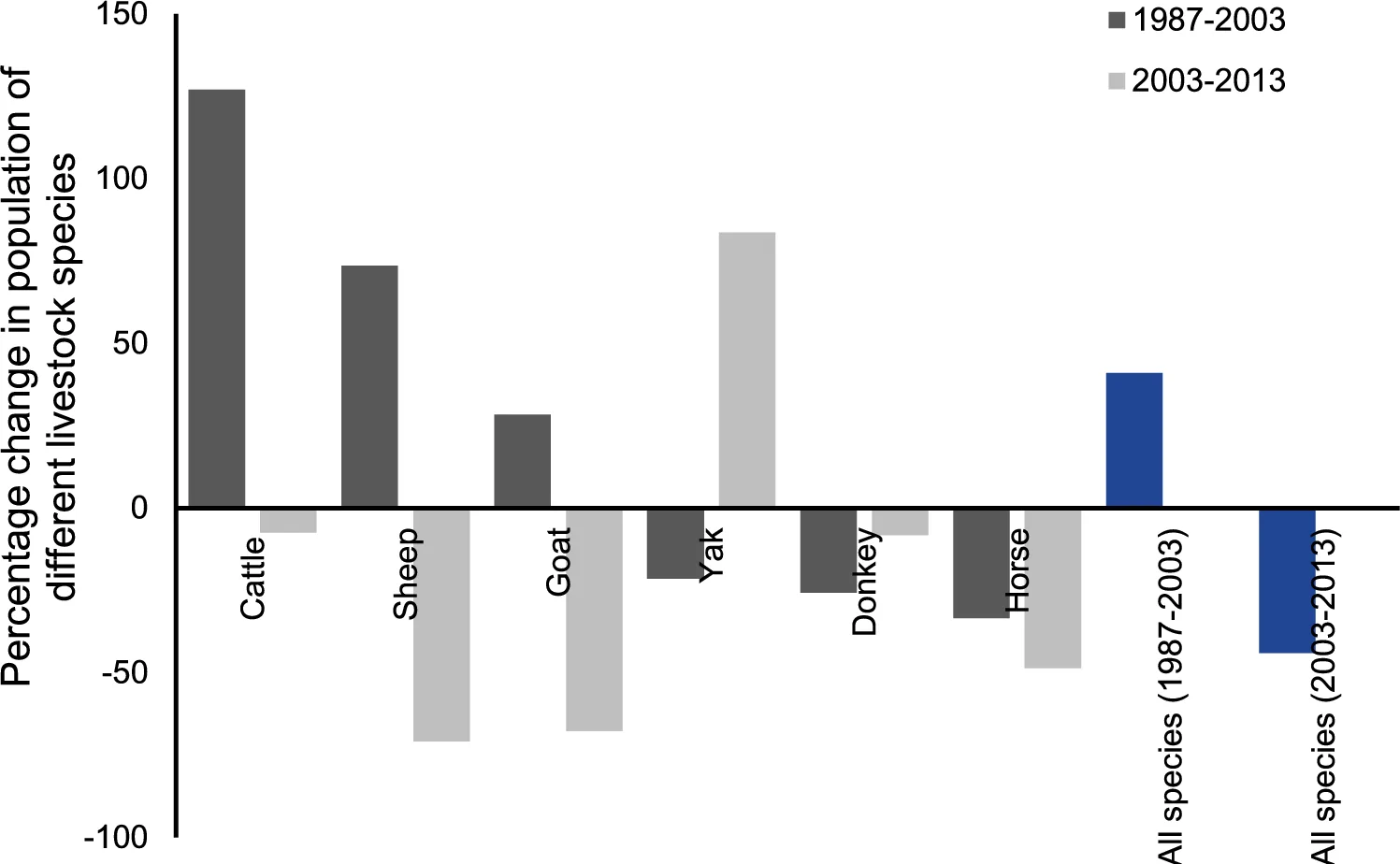
Population change across different livestock species between 1987–2003 and 2003–2013 across 25 villages in the Upper Spiti Landscape. Note: Cattle include yak-cow hybrids as well. *Source*: Census data for 1987 and 2003 (courtesy Nature Conservation Foundation) and information from damage census data for 2013

The population of horses has been declining and has continued to decrease over the last decade (Fig. 2). While the horse and donkey populations declined between 1987 and 2013, the cattle population (including yak-cow hybrids) increased from 1987 to 2003, followed by a 7% decline over the next decade (2003–2014). Post 2003, the population of yaks (particularly yak females) has increased in the landscape (Fig. 2).

**Fig. 3:**
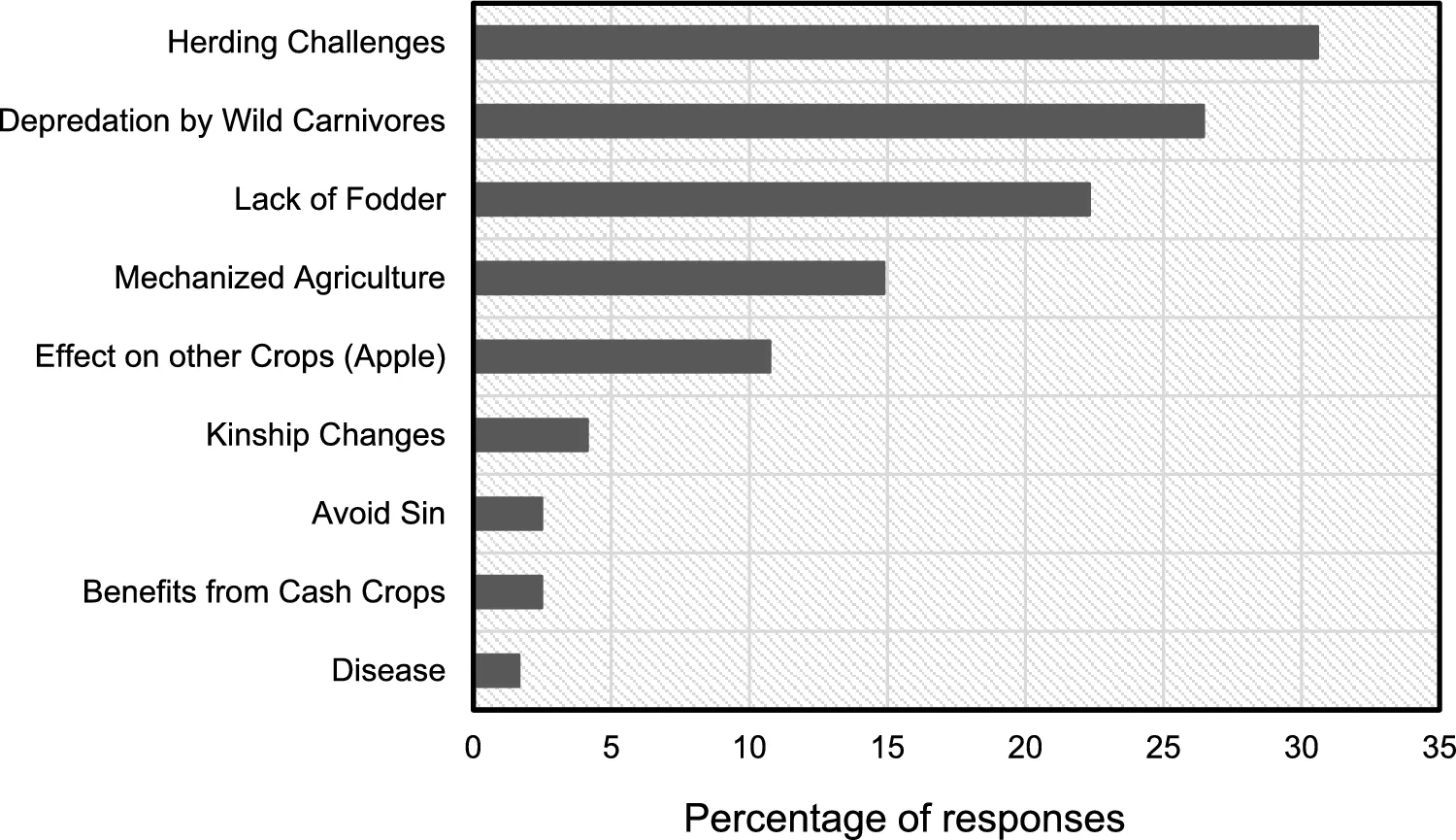
Percentages of responses for causes of livestock decline mentioned by survey respondents who gave an affirmative response for depredation by dogs as a reason

Although respondents gave multiple reasons for livestock decline in the last decade, livestock depredation by dogs was one of the frequently stated reasons for decline (66% of the responses), followed by herding challenges (38% of the responses), lack of fodder (28% of the responses) and depredation by wild carnivores (wolf and/or snow leopard) (25% of the responses). Other reasons for decline (< 15% of the responses) included mechanized agriculture, adverse effects of livestock (specifically sheep and goats) on other crops (apple saplings), kinship changes,^2^ higher cash income from green peas, and diseases. We plotted the reasons for decline stated by respondents who reported livestock depredation by dogs (Fig. 3), essentially demonstrating the difficulty in disentangling these causes. For example, reasons such as “herding challenges” and “lack of fodder” have been represented as two themes, as the former emphasizes the practice of herding. However, fodder is also an important aspect of herding itself. Similarly, both dogs and depredation by wild carnivores add to the larger herding challenges as well. For those who stated that there was an increase in livestock populations (10% of the respondents), increased economic benefits (from large and medium-bodied livestock), and manure requirements due to increased access to agricultural land (through government schemes) were stated as the reasons.

**Fig. 4:**
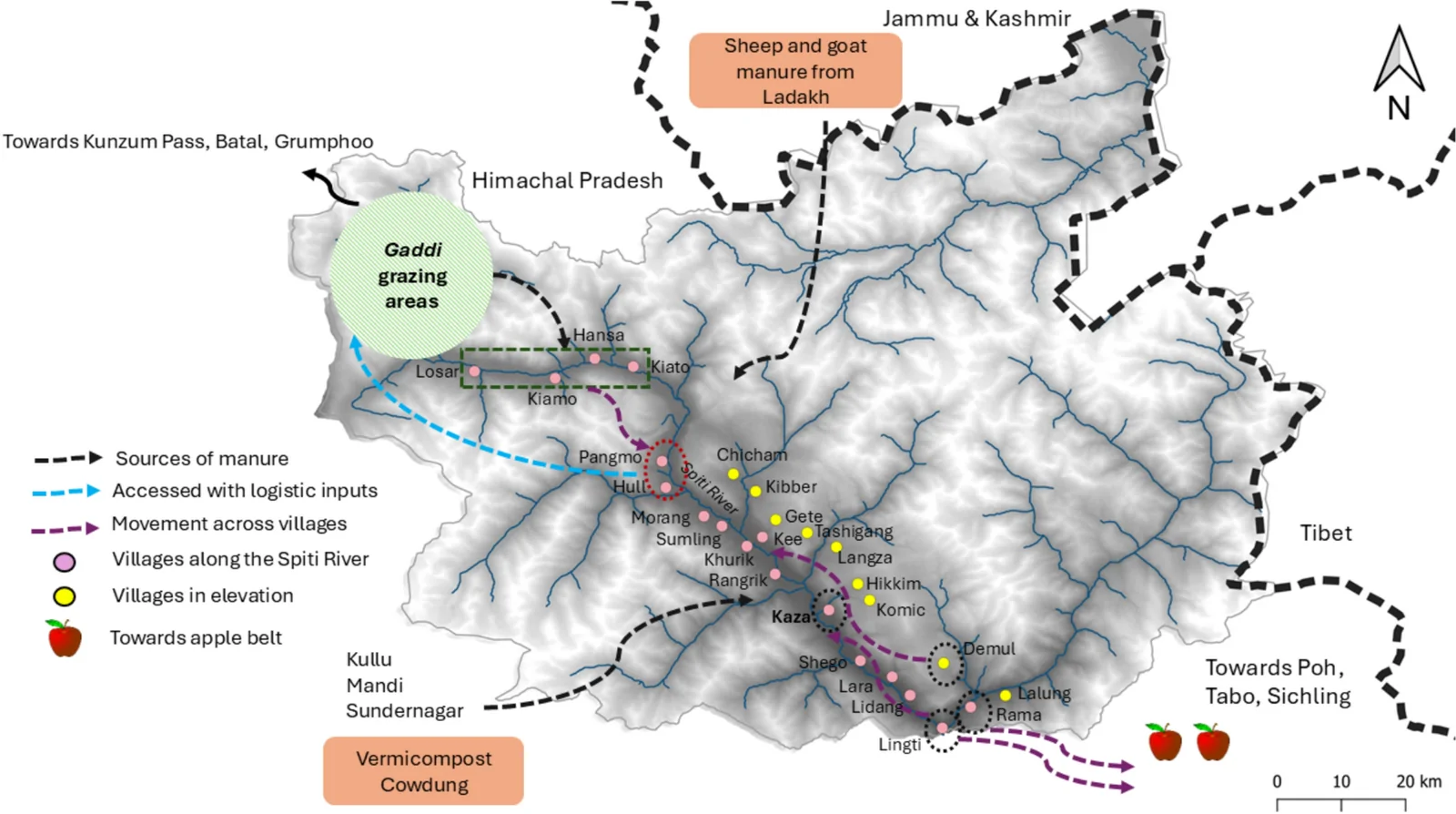
Digital Elevation Model (DEM) of the study area depicting sources of manure accessed from different locations within the Upper Spiti Landscape. Darker areas of the map depict lower elevations while lighter areas depict high elevation zones. Villages marked in the green box are those that have access to manure from the *Gaddis* (migratory pastoralists). Some key villages from where manure is transported to other villages are marked in hashed circles

### Manure inputs for agriculture

About 44% of the respondents mentioned accessing manure outside the landscape. Of these, nearly 38% of the respondents accessing manure belonged to a single panchayat (Lossar) (see Fig. 4 for a visual representation of manure movement within the landscape). The residents of this panchayat have access to sheep and goat manure through a grazing pact with the trans-human *Gaddi* pastoralists. The *Gaddis* arrive in the summer months (May–August) from lower elevation regions (Kullu, Kangra, etc.) in search of fresh pastures for their herds. The *Gaddis* pay the local village council a remuneration to access village pastures, and residents can collect the manure from these areas. If residents need to access manure from *Gaddis* outside village pastures, they pay a nominal fee along with the costs of transporting the manure. Most of these transactions are based on personal liaisons established over the years between the migratory pastoralists and the residents. Though residents of Lossar panchayat have been acquiring manure for over two decades, the respondents mentioned an increase in the quantum of manure acquired over the last 5–6 years. Over the years, a few other villages, such as Hull and Pangmo, have started accessing areas beyond the Kunzum Pass (between Batal and Grumphoo) to collect manure from the areas used by migratory pastoralists.

However, a larger proportion of the respondents (62%) has been accessing different varieties of manure (mainly cow dung manure and now vermicompost) from places outside Spiti, such as Kullu, Mandi, and Sundernagar. Traders from Ladakh also occasionally sold manure from small livestock. With the growth of apple orchards lower down in the Spiti Valley (Poh, Tabo, and Sichling), people in these villages stopped rearing small-bodied livestock to prevent crop damage. Residents sold or moved their sheep and goats to villages in Upper Spiti (Demul, Rama, and Lingti) through an agreement (*doksa*) to supply manure for the orchards. In one of the villages (Lingti), residents lacked land for agriculture and had only small livestock to sell their manure to other villages. Manure was sold at USD 5.5 (~ INR 350) for 40 kg in 2014–2015. Some villages (in the elevations) collected cattle and horse dung from pastures. The village council, or *goba*, regulated dung collection; however, much of the dung collected was used for firing household stoves during peak winters, while the amount used in agriculture was minimal.

### Perception of livestock damage by dogs and future livestock preference

Nearly 59% of respondents experienced livestock depredation by dogs in the last 5 years. For villages higher up in the valley, which have small-bodied livestock, respondents mentioned continuous predation pressure from dogs. Nearly 48% of the respondents considered dogs a “high” threat for their livestock and perceived them to be more damaging than wild carnivores. Close to half of the respondents (42%) did not want to keep small-bodied livestock, while a quarter were unsure about keeping them in the next 10 years. Only a third of the respondents expressed a desire to continue keeping small-bodied livestock in the future. For the respondents who were unsure about rearing small-bodied livestock in the future, the number of households in the village that maintained sheep and goats in the future had a substantial bearing on their decision.

## Discussion

Globally, mountain societies and their production systems have been experiencing significant transformations due to political, socio-economic, and climatic factors (Namgail et al. 2007; Singh et al. 2013; Kerven et al. 2016; Pandey et al. 2017; Singh and Kerven 2023). In general, livestock population declines, and changes in traditional practices have been reported for pastoralists in Himachal Pradesh as well as in other high-altitude landscapes (Namgay et al. 2014; Pandey et al. 2017; Ghoshal et al. 2023; Ladon et al. 2023; Sheth and Saberwal 2023).

**Fig. 5:**
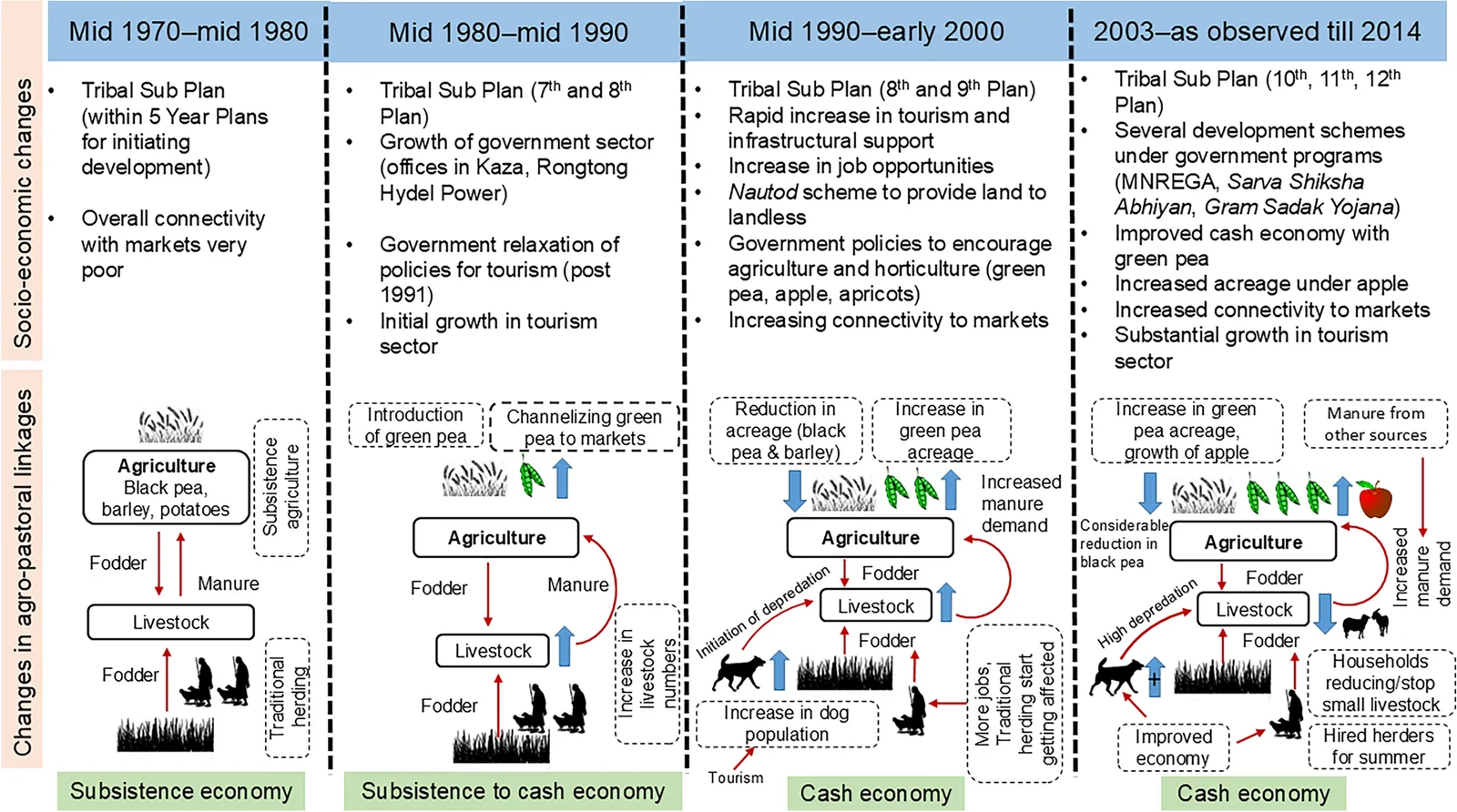
Schematic view of socio-economic changes and changes in agriculture and pastoral practices in the Upper Spiti Landscape in the last three decades

The last three decades in Spiti have experienced a series of socio-economic changes (Mishra 2000; Mishra et al. 2003; Anonymous 2011). The consequences of these changes are visible not only in traditional agricultural and pastoral practices, but also in the emergence of new problems such as an increasing dog population, persistence of livestock depredation by dogs, a decreasing trend in livestock numbers (specifically small-bodied livestock), and growing demands of a cash-crop economy impacting existing linkages (Fig. 5).

### Changes in livestock population and perceived reasons for change

Although the overall livestock population in the Upper Spiti Landscape increased from 1987 to 2003, it decreased by 44% after 2003, with small-bodied livestock specifically contributing to this decline. The declining trend of the livestock population was also supported by our data through interview surveys. Previous studies (a subset of villages in the same landscape) have reported a similar trend as well (Singh et al. 2015, 2020). The landscape has seen a significant decline in the population of *Chumurti* horses, which started a decade ago. However, in recent years, village councils of a few villages (such as within the Kibber panchayat) have mandated that farmers with larger land holdings (*Khangjen*) maintain horses for cultural purposes (such as during barley harvest, and *Losar* or New Year) where horses play an essential role in the ceremonies.

Populations of female yak-cow hybrids (*dzomo*) and cattle were maintained at relatively high numbers with only a minimal decline in the last decade (2003–2013). A dairy loan initiative (*Doodh Ganga Yojna*) by the state government, launched in 2009, has encouraged the milk economy, helping people meet the demand for milk in hotels, restaurants, and schools in Kaza and Rangrik, along with a concomitant demand for manure. The landscape witnessed an increase in the number of yaks (especially yak females). The yaks are free-ranging in pastures for most of the summer, and the sale price for these animals ranged between USD 780–1100 for adults and USD 160–240 for calves in 2014. Although severed trade relations with Ladakh initiated the breeding of yaks in Spiti (Mishra et al. 2003), the ease of managing yaks and the economic benefits derived have increased their population.

While tourism has been a promising means of employment for locals, resulting in increased income levels, the rapid growth of this industry in fragile environments can have a serious impact on long-term sustainability (Geneletti and Dawa 2009; Fan et al. 2015). More importantly, when several interventions interact (e.g., tourism, government policies to enhance livelihoods), the repercussions may be acute, as seen in the increased population of free-ranging dogs and the problem of livestock depredation in the landscape (Home et al. 2017). Although socio-economic changes led to a decline in the population of small-bodied livestock, resulting in the attrition of traditional herding arrangements, respondents reported that increased predation by domestic dogs from 2003 to 2013 has made it challenging to maintain sheep and goat populations in the long term. Although depredation by dogs was reported to be the most frequent cause of livestock decline, other reasons mentioned, such as herding challenges, lack of fodder, and depredation by wild carnivores, demonstrated overlaps, indicating that they may not be mutually exclusive.

Although the livestock sector has been highly dynamic globally (Thornton 2010), studies show a localized decline in livestock populations across high-altitude Himalayan agro-pastoralist communities (Upadhyay 2020; Ladon et al. 2023). A recent study on the Qinghai Plateau demonstrated a similar trend to that observed in the landscape. A comprehensive analysis of livestock populations over a 30-year period revealed a notable decrease between 2004 and 2014. The number of yaks significantly increased, while the numbers of sheep, goats, and horses markedly decreased (Xi et al. 2024).

### Livestock changes and their effects on agriculture

Integrating agriculture with livestock production is one of the most sustainable forms of farming in agro-ecosystems (Lemaire et al. 2014). Green pea cultivation relies on manure inputs from two sources: compost from dry pit toilets (*Chhaksa*) and livestock manure. Since it is strongly associated with organic manure quality and availability, changes in livestock population can affect long-term links. The increase in green pea acreage between 1987 and 2003, driven by market demand, government incentives (*Nautod* scheme), and changes in traditional agricultural practices (reduced fallow rotation) (Anonymous 2011; Singh et al. 2015), may have facilitated an increased demand for manure and subsequent rise in livestock population. However, since 2003, the decline in livestock populations, especially small-bodied livestock, has reduced the local supply of manure for agriculture. The residents of the Lossar panchayat reported that larger quantities of manure have been extracted from the pastures used by the *Gaddis* over the last few years. The growth of apple orchards in the south-eastern part of the landscape has led to an outflux of small-bodied livestock to protect apple saplings, but a demand for their manure from neighboring villages. The residents identify gradation in manure quality across the different livestock species, indicating a greater preference for sheep and goat manure. Small-bodied livestock, being fore-gut fermenters, produce high-quality manure compared to the larger hind-gut fermenters, such as donkeys and horses, which produce copious amounts but relatively poor-quality manure (Batzli and Hume 1994). In recent years, the demand for vermicompost has increased due to its recognition as a valuable organic source of manure for agriculture and horticulture (Chaudhary et al. 2004; Mosa et al. 2015), alongside farmyard manure.

### Livestock depredation by dogs, challenges in herding, and possible future trajectories

The residents of the Upper Spiti landscape have been facing significant economic losses due to the presence of domestic dogs (Home et al. 2017). The socio-economic changes and subsequent attrition of traditional herding patterns created an increasing demand for hired labor (Singh et al. 2015), facilitating the movement of migrant communities from Bihar, Uttar Pradesh, and Nepal during the summer months. These laborers are hired to accompany the main herder (*Lugzi*). The *Lugzi* is assisted by the *Rayok* (for herding sheep and goats), a member of a household with small-bodied livestock. The role of *Rayok* is on a rotational basis. For villages where the number of households maintaining small-bodied livestock is reduced, the turn to assist is repeated at shorter intervals, increasing herding difficulties at the individual household level. In recent years, migrant labor, especially children, has been engaged in herding livestock, which has affected vigilance levels in this practice (Home et al. 2017).

Along with the existing challenges, livestock depredation by dogs has added to herding challenges, particularly for small-bodied livestock. Since 2015, there has also been an emerging trend of larger livestock species, such as yak calves, being targeted by dogs (pers. comm). With the reduction in small-bodied livestock, this shift in prey may be exacerbated in the coming years.

Singh et al. (2020) also noted erosion in Traditional Ecological Knowledge (TEK) in the context of livestock resource management, specifically among the younger generation. Environmental changes will also likely transform this economy if the apple belt shifts further upstream due to climate warming.

**Fig. 6:**
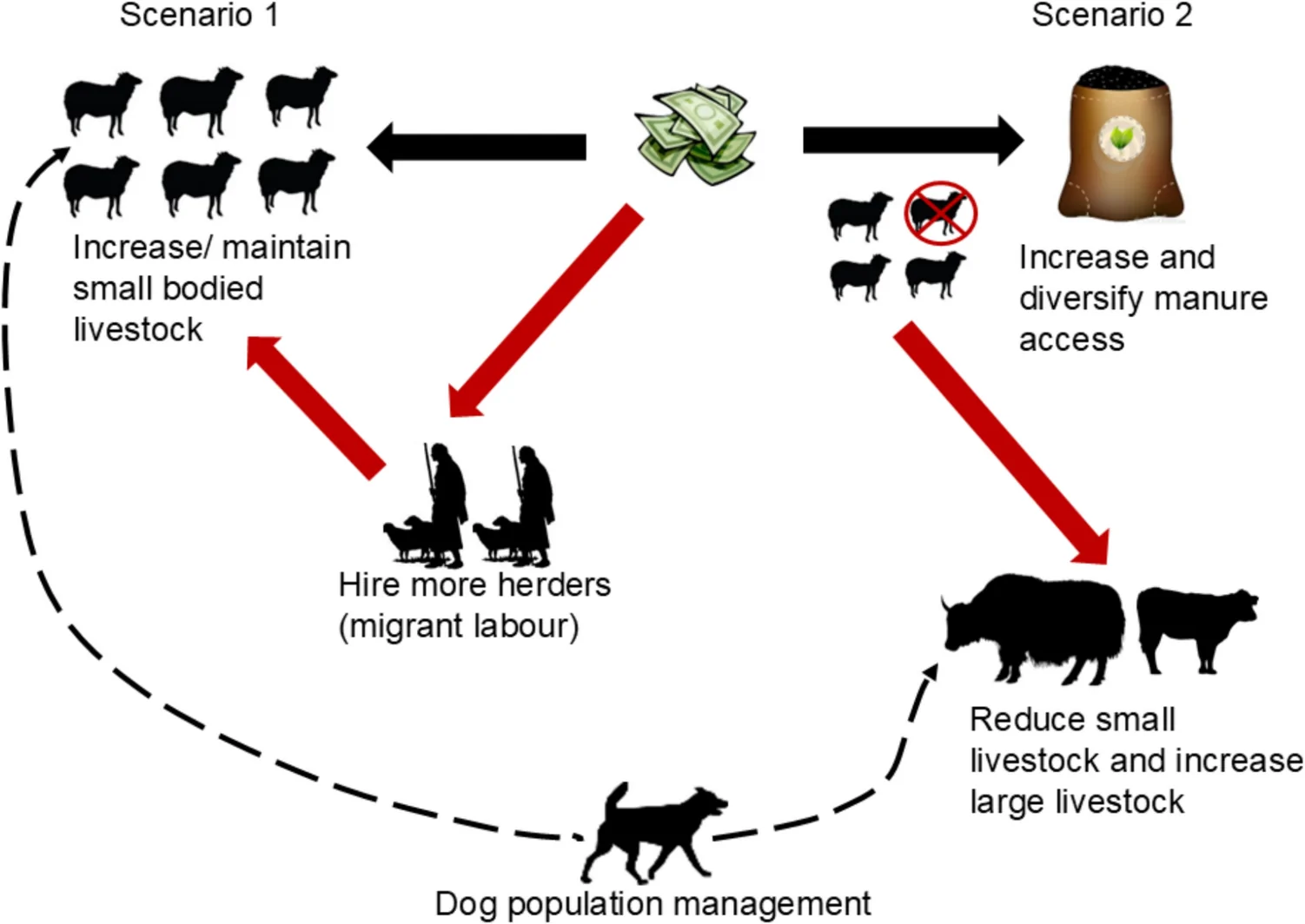
Possible future trajectories in livestock population and management as hypothesized for the Upper Spiti Landscape

We envision two possible trajectories for how these agro-pastoral links could evolve in the coming years, depending on livestock population management (Fig. 6). In scenario 1, we hypothesize that higher income potential among resident agro-pastoralists will enable hiring herders, thereby maintaining or increasing the number of small-bodied livestock. In scenario 2, the cash income will help diversify and increase access to manure from outside sources. The residents will also reduce small-bodied livestock and increase large-bodied livestock, such as yaks and cattle, for ease of herding and greater economic benefits. Although the changes in the cash economy may increase or decrease small-bodied livestock under either scenario, the impact of domestic dogs remains visible in the absence of a systematic dog population management program.

It is worth noting that these scenarios are not mutually exclusive and often coexist with some variation. While hiring herders during the summer is common across most villages, those in the valley have generally stopped herding small-bodied livestock and have increased their herds of large-bodied livestock. Residents of the valley also have access to a more diverse range of manure sources. Villages at high altitudes still maintain small-bodied livestock, although the number of households with small stock has substantially decreased. Being slightly more remote, these villages may have to maintain small-bodied livestock to meet their manure requirements for agriculture. Though in scenario 1, there is a likelihood of hiring more herders to retain/increase small-bodied livestock, the availability of migrants for herding could be a potential limiting factor. As mentioned, this does not solve the problem of high depredation by domestic dogs, which requires concerted and multi-agency action.

An important caveat to this study is the capture of data for the timeline 2003–2013; therefore, the perceptions recorded may have changed in the present times. However, we would like to reiterate the decreasing trend of livestock within the landscape, specifically for the small-bodied livestock mentioned in subsequent publications (Anonymous 2018; Singh et al. 2020), which were published after the data collection for this study. Recent studies in the landscape have shown how wildlife species are reshaping their temporal niches and behavioral patterns to accommodate living with dogs, indicating the continued impacts of domestic dogs in the study area (Justa and Lyngdoh 2023, 2025; Ramesh et al. 2025).

## Conclusion

Livestock holding and composition are influenced by various local and global factors (Mishra et al. 2003; Rahman et al. 2008; Singh et al. 2013; Bai et al. 2021; Sheth and Saberwal 2023). From 2003 to 2013, Spiti has seen changes in livestock composition and numbers owing to (a) depredation by dogs, (b) human resource needs, (c) availability of migrant labor to herd livestock, (d) market influences, and (e) cultural reasons.

Himalayan mountain systems are complex socio-ecological spaces, and their continued well-being is crucial to maintain the respective capacities of the ecosystem and the livelihoods dependent on it. While diversification of production systems (agriculture and livestock) helps minimize risks, their interactions with external factors (such as tourism and markets) can result in emergent properties with unpredictable feedback loops in socio-ecological systems (Walker et al. 2004).

Effective dog population management requires a concerted effort from multiple agencies (Lambertucci et al. 2024). Domestic dogs pose not only a threat to livestock but also impact other wildlife species within the landscape (Ghoshal et al. 2016; Justa and Lyngdoh 2023, 2025) through direct interactions, thereby increasing the risk of disease transmission (Home et al. 2022). In the Upper Spiti landscape, one key management intervention that should be enforced is garbage control, particularly during the tourist season, as reducing access to food subsidies is effective in regulating overabundant carnivores within a short period (Bino et al. 2010). While some efforts have been made to enclose garbage dumps to reduce access (Bijoor 2016), managing the domestic dog population within the landscape requires government and non-government agencies to work in tandem. Although animal birth control (ABC) is the most preferable means of dog population control in India, the current strategy of sterilization in the landscape is unlikely to yield the desired results, mainly because of the low numbers being neutered, along with the complication of inaccessible dogs that are never caught.

In the Trans-Himalayan arid landscape of the Upper Spiti Valley, the interactions between traditional livelihoods and external market-driven economics have produced an unlikely keystone player: the domestic dog, whose impacts have been disrupting intricately linked production systems. The impact of dog depredation on livestock will continue to be an essential driving factor, unless broader institutional mechanisms are implemented to manage this issue within the landscape.

## Supplementary Information

Below is the link to the electronic supplementary material.

Supplementary file1 (DOCX 118 KB)

## Data Availability

The data supporting this study’s findings are not openly available due to reasons of sensitivity and are available from the corresponding author upon reasonable request.

## Footnotes

1 www.census2011.co.in.

2 *Khangjen to Khingjung*; The social system in Spiti is characterized by a primogeniture system of inheritance where the eldest son inherits the land and property and becomes the Khangjen. If the family is divided and stays in separate households, they are referred to as Khingjung.
