# Supplementary material for: Shifting linkages: Agro-pastoralism changes in the Upper Spiti Landscape and the emerging role of free-ranging dogs
Source: Ambio. 2026 May 3;55(11):2780–91. doi: 10.1007/s13280-026-02390-2 (PMC13661835; doi:10.1007/s13280-026-02390-2)
Supplement: Supplementary file 1 [file 13280_2026_2390_MOESM1_ESM.pdf]

Supplementary Material. This Supplementary Information has not been peer reviewed.

Title of Manuscript: Shifting linkages: Agro-pastoralism changes in the Upper Spiti Landscape and the emerging role of free-ranging dogs.

Journal Name: Ambio

List of Authors:

Chandrima Home[1, 2, 3]

Siddhartha Krishnan[2]

Yash Veer Bhatnagar[4,5]

Abi Tamim Vanak[2,6]

Corresponding Author: Chandrima Home[1, 2, 3]

Email of corresponding author: chandrima.home@manipal.edu

Author Affiliations:

1. Shristi Manipal Institute of Art, Design and Technology, Manipal Academy of Higher Education, Manipal, India
2. Ashoka Trust for Research in Ecology and the Environment, Bengaluru.
3. Centre for Doctoral Studies, Manipal Academy of Higher Education, Manipal, India
4. International Union for Conservation of Nature, New Delhi
5. Nature Conservation Foundation, Mysore
6. School of Life Sciences, University of KwaZulu-Natal, Durban, South Africa

Contents of Supplementary information:

1. Semi-structured questionnaire survey used for interviews

Respondent Information:

1. Respondent ID
2. Age
3. Gender
4. Education
5. Current livestock holding
6. Livestock holding 5 years ago
7. Livestock holding 10 years ago
8. Land holding (in bighas)
9. Anyone with govt. job
10. Number of members in the family

Livestock-agriculture relationships:

1. Nature of change of livestock: Increase/Decrease/No change
2. What are the reasons for livestock change (if any)? (open ended)
3. What are the pros and cons of livestock change (if any)? (open ended)
4. What are the effects of livestock change (if any) on agriculture (say in terms of manure)? Do you procure/sell manure? If so from where? (open ended)
5. What would be the livestock composition in the next 10 years to come? Would you still have the same composition as now? If yes/no then why? (open ended)

Human-dog relationships:

1. Do you currently own a dog?
2. Any history of having dogs as pets?
3. Do you think there has been a change in the dog population? If so then what changes and the reasons for change?
4. Any experience of livestock depredation? If so when? Information obtained for past experience (5-10 years) and present (in the last 5 years)
5. Are these depredation by resident or transient dogs?
6. Have you observed dogs harassing/attacking people in your village?
7. Any personal experience of harassment by dogs?
8. Any intervention for dog population management?
9. How will you rank dogs vis a vis native carnivores in terms of threat to livestock? (on a scale of -1, 0, 1
10. Any experience of livestock depredation by snow leopards and wolves?

Attitude questions for dogs: (Ranked Data)

1. How do you feel about dogs? Why do you feel so?
2. Would you want dogs in and around the village? If yes then why?
3. Do you feed dogs in your village? If yes/no then why?
4. How will you rank dogs vis a vis native carnivores?
5. What would you do to protect your livestock from dog predation?
